# 

**Published:** 1905-11-15

**Authors:** 


### ANNOUNCEMENTS.

THE DETROIT DENTAL SOCIETY.—This society held its first meeting for the coming season, October 12th, which was well attended. A good paper was read by Dr. G. F. Burke, on Business Methods. The society has issued its program for the next eight months, and there is little doubt but its meetings will be interesting. The society now has over one hundred members. We shall begin the publication of its proceedings in the January issue of the REGISTER.

PROF. W. D. MILLER.—Prof. Miller and his wife spent the months of September and October visiting in this country. Dr. Miller is not now practicing, but devotes his entire time to his educational and scientific work. He enjoys athletic diversions, particularly a game of football or base ball, and is himself an expert golf player, holding the championship of Germany and Austria. His daughter married last year and lives in Middletown, Conn., this makes it probable that we shall see him often.

DR. CAROL M. MCELROY has removed from Delaware, Ohio, to New York City and will engage in the general practice of dentistry at No. 70 West 49th Street. The REGISTER readers in Ohio will wish her abundant success.

OHIO STATE DENTAL MEETING.—The fortieth annual meeting will be held in Columbus December 5th, 6th and

7th, at the Great Southern Hotel. An exceptionally strong program of papers and clinics has been provided, and it only needs a large attendance to assure a profitable meeting. For full information write to Dr. F. R. Chapman, Secretary, Schultz Building, Columbus, Ohio.

—✦—

OFFICERS NORTHERN INDIANA SOCIETY.—At Logansport, September 19th and 20th, the following officers were elected: President, Dr. Otto U. King, of Huntington; Vice President, Dr. F. M. Bozer, of Logansport; Secretary, Dr. S. A. Bell, of Hammond; Superintendent of Clinics, Dr. L. A. Salisbury, of Crown Point. The next meeting will be held at Hammond.

—✦—

DR. JOHN LITTLEFIELD died in Philadelphia, July 13th, in the eighty-second year of his age. He began the practice of dentistry in 1845 at Milton, Mass., In 1868 he entered the dental supply business in Boston, with Codman and Shurtleff. In 1888 he became connected with the S. S. White Dental Manufacturing Co., and remained there until the time of his death. He was well known and a man of force and character. He did much to give his business house its enviable reputation.

—✦—

THE STATE BOARD JOURNAL.—Under this title a new journal is announced from Washington, D. C. It is proposed to discuss all State Board of Examiners problems. The State Boards are coming into notice rather rapidly of late, and it may be necessary for them to have an organ, but we do not believe there is any urgent demand for a monthly publication devoted exclusively to this subject. We fear its subscription list will not reach the 5,000 mark it starts out with, and its editor's copy pump will begin very soon to "suck air," as our friend Barrett used to remark.

LEO T. SAUERBRUN MARRIED.—Dr. Sauerbrun was married September 3rd to Miss Ora Lederer, at New Washington, Ohio. They will reside in Mansfield, Ohio, where the doctor has a nice practice. He graduated from the University of Michigan in 1901.

ANOTHER DENTAL CONGRESS.—There is to be an exposition held at Jamestown, Va., in 1907, and a committee has been appointed by the Virginia State Dental Society to confer with the National Dental Society to organize a dental congress.

DR. HUDSON P. HURD.—Dr. Hurd died September 6th, in Cleveland, Ohio, where he was well known as one of the oldest practitioners in the State. He was eighty-five years of age when he died. He had not been in active practice for several years. It is said that he was the first man to administer nitrous oxide gas in Cleveland.

THE AMERICAN SOCIETY OF ORTHODONTISTS.—This Society held a very interesting meeting at the Stratford Hotel, in Chicago, September 28th to 30th. There was a large attendance and the program was very interesting, and instruction in papers, clinics and exhibits of models and reports of cases. Dr. R. Ottolengui, of New York, was elected President, and Dr. Anna Hopkins, of St. Louis, was re-elected Secretary.

SECOND VOLUME, TRANSACTIONS OF INTERNATIONAL CONGRESS.—The second volume of the transactions has just been received and contains the proceedings of the sections on Chemistry and Metallurgy, Oral Hygiene, Materia Medica and Therapeutics, Oral Surgery and Orthodontia. It is well printed and bound like its predecessor. The orthodontia section has fifteen papers and addresses; the surgical section ten papers and addresses; the therapeutic section has eighteen papers and addresses, and the chemical section has four papers.

FRATERNAL DENTAL SOCIETY CLINIC.—The first annual clinic of this society will be held in the Barnes Dental College, at St. Louis, November 20th and 21st. Special features will be gold filling and porcelain filling. There will be dealer's exhibits. Hotel and railway rates will be in effect.

INSTITUTION OF DENTAL PEDAGOGICS.—The annual meeting of the Institute of Dental Pedagogics will be held in the Fifth Avenue Hotel, New York, December, 28th, 29th and 30th. The following subjects will be discussed:

Anesthesia, Extraction, Operative Technic, Prosthetic Technic, Crown and Bridge Technic, Orthodontia Technic, Porcelain Technic, Chemistry, Anatomy and Oral Surgery, Teaching in the Infirmary.

The main idea of the meeting will be "How should these subjects be presented to a Dental Student." This will be the most important dental meeting of the year, especially for teachers. As far as possible every demonstrator as well as the professors should make an effort to be present.

W. E. WILLMOTT, *Sec'y.*

